# Dihydromyricetin Inhibited Migration and Invasion by Reducing S100A4 Expression through ERK1/2/β-Catenin Pathway in Human Cervical Cancer Cell Lines

**DOI:** 10.3390/ijms232315106

**Published:** 2022-12-01

**Authors:** Min-Chieh Hsin, Yi-Hsuan Hsiao, Pei-Ni Chen, Chiao-Wen Lin, Po-Hui Wang, Shun-Fa Yang, Chung-Yuan Lee

**Affiliations:** 1 Institute of Medicine, Chung Shan Medical University, Taichung 402, Taiwan; 2 National Institute of Cancer Research, National Health Research Institutes, Zhunan 350, Taiwan; 3 Department of Obstetrics and Gynecology, Changhua Christian Hospital, Changhua 500, Taiwan; 4 Women’s Health Research Laboratory, Changhua Christian Hospital, Changhua 500, Taiwan; 5 School of Medicine, Chung Shan Medical University, Taichung 402, Taiwan; 6 Department of Medical Research, Chung Shan Medical University Hospital, Taichung 402, Taiwan; 7 Institute of Oral Sciences, Chung Shan Medical University, Taichung 402, Taiwan; 8 Department of Dentistry, Chung Shan Medical University Hospital, Taichung 402, Taiwan; 9 Department of Obstetrics and Gynecology, Chung Shan Medical University Hospital, Taichung 402, Taiwan; 10 Department of Obstetrics and Gynecology, Chiayi Chang Gung Memorial Hospital, Chiayi 613, Taiwan; 11 Department of Nursing, Chang Gung University of Science and Technology, Chiayi Campus, Chiayi 613, Taiwan

**Keywords:** β-catenin, cervical cancer, DHM, metastasis, S100A4

## Abstract

Cervical cancer has a poor prognosis and is the fourth most common cancer among women. Dihydromyricetin (DHM), a flavonoid compound, exhibits several pharmacological activities, including anticancer effects; however, the effects of DHM on cervical cancer have received insufficient research attention. This study examined the antitumor activity and underlying mechanisms of DHM on human cervical cancer. Our results indicated that DHM inhibits migration and invasion in HeLa and SiHa cell lines. Mechanistically, RNA sequencing analysis revealed that DHM suppressed S100A4 mRNA expression in HeLa cells. Moreover, DHM inhibited the protein expressions of β-catenin and GSK3β through the regulated extracellular-signal-regulated kinase (ERK)1/2 signaling pathway. By using the ERK1/2 activator, T-BHQ, reverted β-catenin and S100A4 protein expression and cell migration, which were reduced in response to DHM. In conclusion, our study indicated that DHM inhibited cell migration by reducing the S100A4 expression through the ERK1/2/β-catenin pathway in human cervical cancer cell lines.

## 1. Introduction

Cervical cancer comprises a malignant tumor of the cervix and is the fourth most diagnosed cancer among women [1,2]. It has two histological types, adenocarcinoma (AC) and squamous cell carcinoma (SCC) [1], of which SCC accounts for 70% of diagnoses [3]. Human papillomavirus (HPV) infection plays a primary role in cervical cancer [4]. HPV causes approximately 90–100% of cervical cancer cases, especially in patients aged <35 years [5]. For metastasis to occur, cancer cells must leave their main sites, circulate in the blood, withstand vascular pressure, adapt to the new cellular environment of the secondary sites, and withstand attacks from immune cells [6,7,8]. Metastasis is the primary cause of death in more than 90% of patients with cancer [9,10,11]. Although cancer metastasis is a primary cause of cancer treatment failure and subsequent death, little is known about it.

S100A4, a member of the S100 family of calcium-binding proteins, was discovered in 1989 and initially named metastasin (Mts1) [12,13]. S100 proteins are involved in numerous cell functions, such as proliferation, differentiation, apoptosis, calcium homeostasis, metabolism, inflammation, and motility [14]. S100A4 has been reported to influence metastasis [15,16,17] by promoting the movement and invasion of existing tumor cells, which leads to invasive metastasis. Therefore, S100A4 is the primary prognostic indicator of numerous types of cancer [18].

Dihydromyricetin (DHM, C_15_H_12_O_8_), a component of *Ampelopsis grossedentata*, is a flavonoid compound [19] that has anti-inflammatory [20], antioxidant [21,22], antihypertensive [23], hypoglycemic [24], hepatoprotective [25,26], anticarcinogenic [27], and antimetastasis effects [28,29,30]. DHM reportedly inhibits invasion and metastasis in hepatocellular carcinoma [31]; DHM also inhibits cell migration in human proliferative vitreoretinopathy cells through the inhibition of MMP-2 expression [30]. However, the molecular mechanism underlying the effects of DHM on cervical cancer remains unclear. Thus, this study examined the effects of DHM with potential antimetastatic properties in in vitro DHM-treated HeLa and SiHa human cervical cancer cells to investigate the signaling pathway of this process.

## 2. Results

### 2.1. Cell Viability and Cell Migration and Invasion of DHM on Cervical Cancer Cell Lines

**Figure 1:**
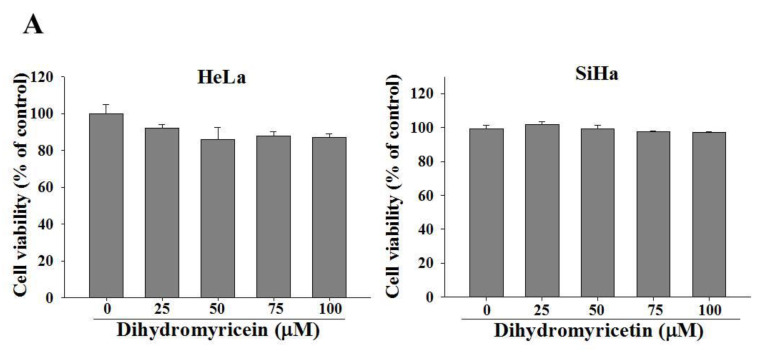

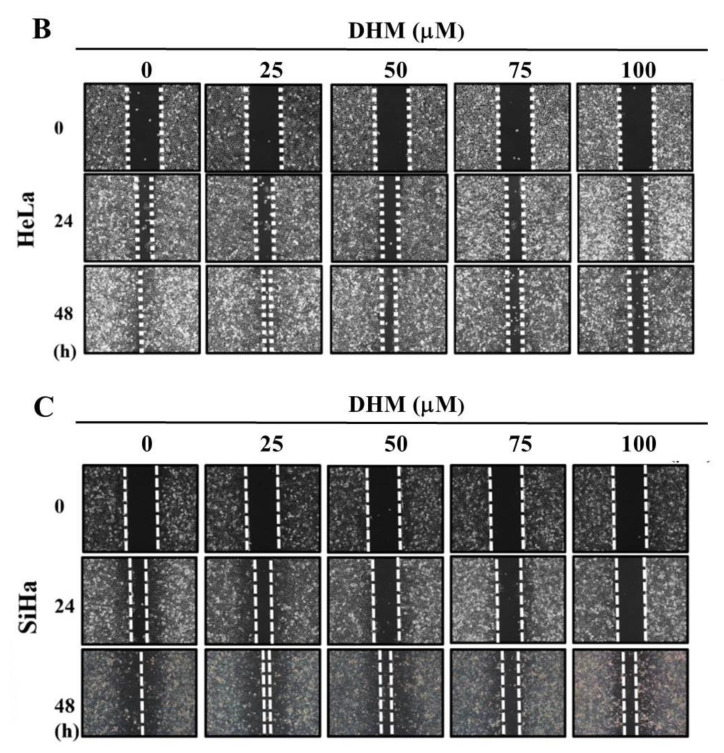
Effects of DHM on cell viability and wound healing assay in cervical cancer cell lines. (**A**) HeLa and SiHa cells were seeded onto 24-well plates and treated with DHM (0, 25, 50, 75, 100 μM) for 24 h and assessed for cell viability. (**B**) HeLa and (**C**) SiHa cells were treated onto 6-well plates and a line was drawn between cells and cells, then observed for the ability of healing in 24 h and 48 h on various DHM concentrations by microscope. Cells were photographed using microscope (100×).

**Figure 2:**
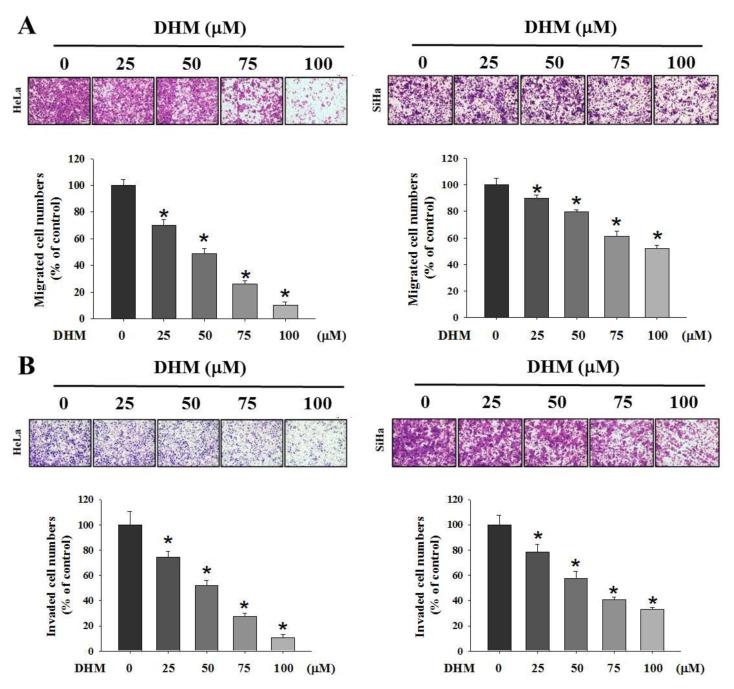
Effects of DHM on cell migration and invasion in cervical cancer cell lines. (**A**,**B**) HeLa and SiHa cells were seeded onto a 6 cm dish and treated with DHM (0, 25, 50, 75, 100 μM) for 24 h. Analyzed by Boyden chamber assay. The values represented the mean ± S.D. from three determinations per condition repeated three times. *, *p* < 0.05 compared with untreated. Cells were photographed using microscope (100×).

We first investigated the effects of DHM on cervical cancer cell viability. HeLa and SiHa cells were treated with 0, 25, 50, 75, and 100 μM DHM for 24 h and were analyzed with an MTT assay. The results indicated no toxic effects on the cervical cancer cells (Figure 1A). To assess the antimetastatic effects of DHM on cervical cancer, we performed wound healing and Boyden chamber assays to determine whether DHM can regulate the migration of cervical cancer cells. In the wound healing assay, the cells were treated with various concentrations of DHM (0, 25, 50, 75, or 100 μM) for 24 and 48 h. The results revealed that DHM inhibited the migration of cervical cancer cells in a concentration-dependent manner (Figure 1B,C). In the Boyden chamber assay, we treated the cell lines with DHM for 24 h; the cervical cancer cell lines were then seeded into the upper chamber (invasion: cells seeded on Matrigel-coated filter; Figure 2A,B). As shown in Figure 2A,B, the results reveal that DHM notably reduced the migration and invasion of the cervical cancer cells.

### 2.2. DHM Reduced S100A4 Gene Expression in Cervical Cancer Cells

**Figure 3:**
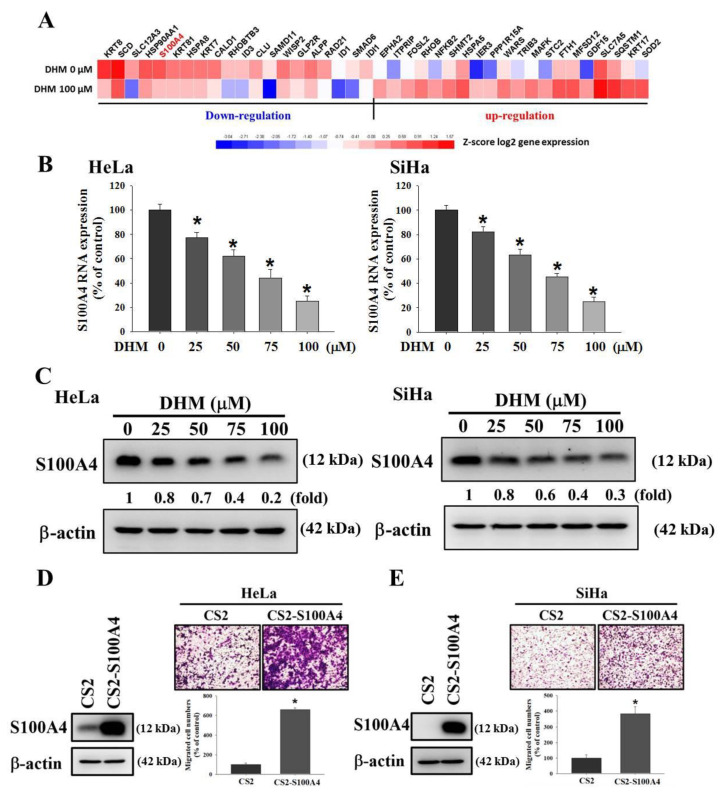
Effects of DHM on S100A4 expression in cervical cancer cell lines. (**A**) Heat map of the hierarchical clustering of 40 differentially expressed genes identified in HeLa cells after treatment with DHM (0, 100 μM). (**B**,**C**) The RNA level and protein level of S100A4 were detected by real-time PCR and Western blotting. (**D**) HeLa and (**E**) SiHa cells were seeded onto 6 cm dish and transfected with CS2-vector or CS2-S100A4. The results were analyzed by Boyden chamber assay and Western blotting. *, *p* < 0.05 compared with untreated. Cells were photographed using microscope (100×).

**Figure 4:**
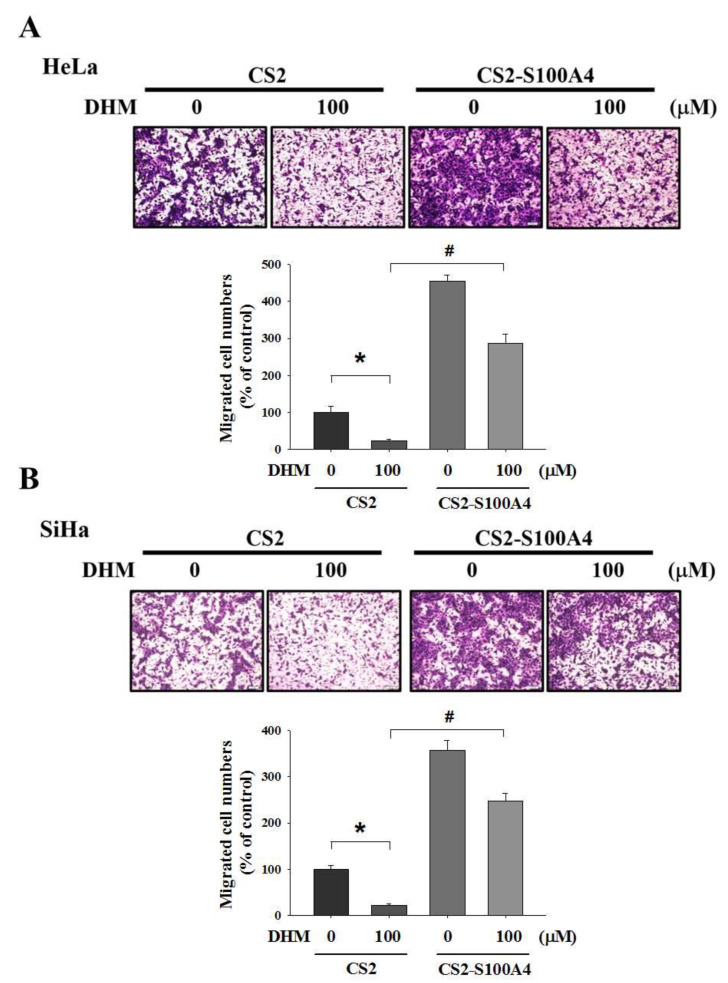
S100A4 overexpression and co-treatment with DHM in cervical cancer cell lines. (**A**,**B**) HeLa and SiHa cells were seeded onto 6 cm dish and transfected with CS2-vector or CS2-S100A4. After 24 h, we co-treatment with DHM (0, 100 μM) then analyzed by Boyden chamber assay (*, *p* < 0.05 compared with untreated; #, *p* < 0.05 compared with CS2-vector with DHM 100 μM). Cells were photographed using microscope (100×).

To identify the target genes that were regulated after being treated with DHM, the RNA-sequencing analysis of the HeLa cell line with DHM (0 or 100 μM) was performed (Figure 3A). As illustrated in Figure 3A, S100A4 is the downregulated gene in DHM-treated cells and there are some reports regarding S100A4 and cancer metastasis [32,33,34]. Therefore, we chose the S100A4 gene as the target gene to investigate its anti-metastatic properties. To validate the RNA sequencing findings of S100A4, we conducted real-time PCR analysis and Western blotting assay and found that DHM inhibited S100A4 expression in cervical cancer cells in a concentration-dependent manner (Figure 3B,C). Subsequently, the Boyden chamber assay indicated that S100A4 overexpression considerably promoted migration in the HeLa cells and SiHa cells (Figure 3D,E). Furthermore, we examined the effect of DHM on the crawling ability of human cervical cancer cells by regulating the S100A4 gene. HeLa cells and SiHa cells were transfected with a CS2-empty vector or CS2-S100A4 for 24 h, then treated with DHM (0 or 100 μM). The Boyden chamber assay results indicated that S100A4 overexpression was mitigated by DHM; that is, S100A4 expression was reduced in both HeLa and SiHa cells (Figure 4A,B). The results indicated that DHM mediates cell migration through the regulation of S100A4 levels in cervical cancer cells.

### 2.3. DHM Inhibited Cell Migration by Reducing S100A4 Expression through ERK1/2/β-Catenin Pathway

**Figure 5:**
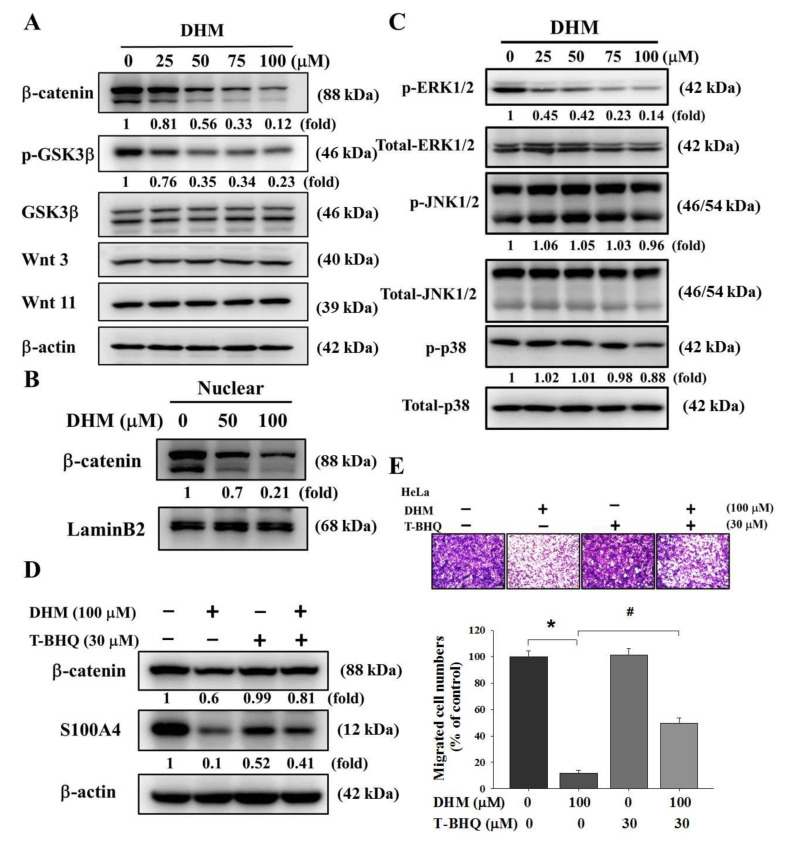
Effects of DHM on β-catenin, p-GSK3β, and MAPK pathway level. (**A**) HeLa cells were seeded onto a 6 cm dish and treated with DHM (0, 25, 50, 75, 100 μM). The results were analyzed by Western blotting. (**B**) HeLa cells were seeded onto a 10 cm dish and treated with DHM (0, 50, 100 μM). The results were analyzed by Western blotting. (**C**) HeLa cells were seeded onto a 6 cm dish and treated with DHM (0, 25, 50, 75, 100 μM) and assessed for the phosphorylation status of ERK1/2, JNK1/2, and p38-MAPK by Western blotting with indicated antibodies. (**D**) HeLa cells were seeded onto a 6 cm dish and treated with DHM (0, 100 μM) for 1h and co-treatment with T-BHQ (30 μM) for 23 h. The results were analyzed by Western blotting. (**E**) HeLa cells were seeded onto a 6 cm dish and treated with DHM (0, 100 μM) for 1h and co-treatment with T-BHQ (30 μM) for 23 h. The results were analyzed by Boyden chamber assay (*, *p* < 0.05 compared with untreated; #, *p* < 0.05 compared with T-BHQ 0 μM). Cells were photographed using microscope (100×).

Dahlmann et al. determined that the abnormal activity of Wnt signal transduction is an early step in cancer metastasis. Furthermore, the metastasis-inducing gene S100A4 was identified as a transcriptional target of β-catenin [32]. Therefore, we conducted a Western blotting assay and found that DHM inhibited the β-catenin expression and GSK3β phosphorylation in cervical cancer cells while DHM had no obvious influence on Wnt 3 and Wnt 11 expression (Figure 5A). Moreover, after the DHM treatment, the nucleation of β-catenin was also inhibited (Figure 5B). We subsequently examined whether DHM could reduce the activation of three major mitogen-activated protein kinases, namely ERK1/2, JNK1/2, and p38. As illustrated in Figure 5C, DHM inhibited ERK1/2 phosphorylation in HeLa cell lines. However, the phosphorylation of JNK1/2 and p38 was not altered by DHM treatment. Moreover, the HeLa cell lines were pretreated with T-BHQ, an ERK1/2 activator, for 1 h, treated with 100 µM DHM for another 24 h, and then analyzed with Western blot assay and Boyden chamber assay. Our results demonstrated that T-BHQ reversed the inhibitory effects of DHM in expressions of β-catenin and S100A4 (Figure 5D) and cell migration (Figure 5E). These findings implicate a causal involvement of the ERK1/2 signaling pathway in the molecular mechanisms underlying DHM-mediated cervical cancer cell migration.

## 3. Discussion

Cervical cancer is a malignant tumor of the cervix, the fourth most diagnosed cancer among women, and the second leading cause of cancer mortality in women aged 20–39 years [35]. Although surgery, radiotherapy, and chemotherapy benefit patients with metastases [36], chemotherapy often has a detrimental effect on patients. Chemotherapy effectively treats cancer, but many patients with cancer are either insensitive or resistant to chemotherapy. Therefore, effective mechanisms that enhance tumor sensitivity to chemotherapy are necessary [37].

DHM is a flavonoid compound that has been widely studied in the food and medicine industries. DHM reportedly inhibits metastasis in multiple cancers, including hepatocellular carcinoma, and human proliferative vitreoretinopathy cells. Furthermore, DHM induces apoptosis and reverses multidrug resistance in ovarian cancer cells through the downregulation of survivin [38]. Fan et al. indicated that DHM promotes autophagy and apoptosis through ROS-STAT3 signaling in head and neck SCC [39]. The present study provided additional evidence for the inhibitory effect of DHM on the cell migration of human cervical cancer cells. DHM also reduced the expression of the S100A4 protein level. S100A4 is an oncogene in several cancers. In glioblastoma, S100A4 is a novel marker, regulator, and critical upstream regulator of the mesenchymal transition [40]. Moreover, S100A4 might induce tumor progression through the stimulation of angiogenesis [41]. S100A4 accelerates tumorigenesis and the invasion of human prostate cancer through the transcriptional regulation of matrix metalloproteinase-9 [42]. We determined that DHM may inhibit the RNA and protein expression of S100A4 in human cervical cancer as well as S100A4 overexpression, which induces cell migration. When treated cells were exposed to DHM and S100A4 overexpression, the overexpression of S100A4 reversed the cell crawling ability inhibited by the DHM (Figure 4).

The molecular structure of the Wnt/β-catenin pathway and its role in signal modulation has been researched extensively [43,44,45,46]. Abnormal activity in Wnt signaling is an early step in the transformation of healthy intestinal cells into malignant tissues, leading to more aggressive tumors and eventual metastasis [32]. In human colorectal cancer, Wnt/β-catenin signaling is a primary signaling pathway [47]. The present study revealed another signaling pathway of DHM regulation: ERK1/2/β-catenin. Yamaguchi et al. indicated that the AKT, ERK1/2, and IKK signaling pathways regulate FOXO3 and β-catenin [48]; these results are consistent with those of our study. When we combined DHM and the ERK1/2 activator T-BHQ in the HeLa cell line, cell migration and the expressions of S100A4 and β-catenin were reversed. Our results indicated that DHM regulated S100A4 gene expression through the ERK/β-catenin pathway, thus inhibiting cell migration. Moreover, a study reported that S100A4 is a direct transcription target of the Wnt/β-catenin/TCF-mediated signaling pathway. The use of new therapeutic interventions or screening of pharmacologically active compounds is strongly recommended to reduce the expression of S100A4 in colorectal cancer [32]. The results of our study indicated that DHM inhibited the expression of translocated β-catenin into nuclear β-catenin. We surmised that DHM regulated the level of β-catenin that translocated into nuclear to target S100A4.

## 4. Materials and Methods

### 4.1. Cell Lines and Culture

Human HeLa and SiHa cervical cancer cell lines were cultured in Dulbecco’s modified Eagle’s medium (Gibco-BRL, Gaithersburg, MD, USA) supplemented with 10% fetal bovine serum (FBS; HyClone Laboratories, Inc., South Logan, UT, USA) and 100 ng/mL each of penicillin and streptomycin (Sigma, Aldrich Corporation, St. Louis, MO, USA) as previously described [49]. All cell lines were cultured at 37 °C in a humidified atmosphere of 5% CO_2_.

### 4.2. Cell Viability Assay

The HeLa and SiHa cells were seeded onto 24-well plates and incubated overnight. Subsequently, cell viability was assessed using a 3-(4,5-dimethylthiazol-2-yl)-25-diphenyltetrazolium bromide assay as previously described [50].

### 4.3. Wound Healing Assay

The HeLa and SiHa cells were seeded onto 6-well plates and incubated overnight. Subsequently, the cells were scratched using pipette tips. We observed cell healing at various time points through microscopy [51].

### 4.4. Quantitative Real-Time PCR

Total RNAs were isolated from SiHa and HeLa cells by using the Total RNA Mini Kit (Geneaid Biotech Ltd., Sijhih City, Taiwan), and cDNAs were reverse transcribed from isolated total RNA by using the High Capacity cDNA Reverse Transcription Kit (Applied Biosystems, Foster City, CA, USA) [52]. SYBR primers used were as follows: S100A4 sense 5′-GAT GAG CAA CTT GGA CAG CAA-3′, antisense 5′-CTG GGC TGC TTA TCT GGG AAG-3′.

### 4.5. Cell Migration and Invasion Assay

We collected the cells using trypsin–ethylenediaminetetraacetic acid (Gibco), and the tumor metastasis assay in vitro was conducted with the Boyden chamber (Neuro Probe, Cabin John, MD, USA) [53]. Treated cells in a 0% FBS medium were loaded into the upper well of the chamber and incubated for 24 h (migration) or 48 h (invasion) at 37 °C. The invasion membrane filters were coated with 10 μL Matrigel (25 mg/50 mL; BD Biosciences, San Diego, CA, USA) and air dried for 5 h in a laminar flow hood. The migration cells were fixed using methanol, stained with Giemsa, and counted using light microscopy.

### 4.6. Western Blot Assay

Total cell lysates were collected with 100 μL of lysis buffer (50 mM Tris-HCl, pH 7.5, 0.5 M NaCl, 5 mM MgCl_2_, 0.5% Nonidet P-40, 1 mM phenylmethylsulfonyl fluoride, 1 μg/mL pepstatin, and 50 μg/mL leupeptin) on ice. After being centrifuged at 13,200× *g* at 4 °C for 30 min, the protein lysates were separated using 10% agarose gel, and transferred onto a nitrocellulose membrane [54]. They were then blocked with 5% nonfat milk in Tris-buffered saline (20 mM Tris, 137 mM NaCl, pH 7.6) for 1 h at room temperature and overnight with first antibodies at 4 °C and second antibodies for 1 h at room temperature.

### 4.7. CS2-S100A4 Transfection

The plasmid of CS2-S100A4 was generously provided by Dr. Isao Matsuura of the National Health Research Institutes. The HeLa and SiHa cells were seeded into 6-cm plates. After being cultured overnight, 5 µg of the empty CS2-vector (GenDiscovery Biotechnology, Taipei, Taiwan) or CS2-S100A4 was transfected into the cells and left for 6 h before the reagent was removed and the cells were cultured with fresh medium overnight.

### 4.8. Statistical Analysis

Significant differences were calculated using the Student’s *t*-test (SigmaPlot 10.0, Jandel Scientific, and San Rafael, CA, USA). Significance was set at *p* < 0.05. The values reported are the means ± standard deviation of at least three independent experiments.

## 5. Conclusions

In conclusion, we discovered that treating cervical cancer with DHM may inhibit cell migration and invasion through the regulation of S100A4 expression through the ERK1/2/β-catenin pathway. DHM regulates the translocating ability of β-catenin through the ERK1/2 pathway, thereby affecting the performance of the target S100A4 and ultimately inhibiting the migration ability of cervical cancer cells. This study presents a new option for restricting S100A4-induced cell motility and metastasis. Thus, DHM may serve as a potential therapeutic target for adjuvant therapy in the future.

## Data Availability

The data presented in this study are available on request from the corresponding author.

## References

[1] Sung H., Ferlay J., Siegel R.L., Laversanne M., Soerjomataram I., Jemal A., Bray F. (2021). Global cancer statistics 2020: Globocan estimates of incidence and mortality worldwide for 36 cancers in 185 countries. CA A Cancer J. Clin..

[2] Hsiao Y.H., Chen P.N., Hsin M.C., Wang P.H., Huang J.Y., Yang S.F. (2021). The risk of distant metastases in patients with gynecologic cancers after surgery: A population-based study. Aging.

[3] Herrero R., Gonzalez P., Markowitz L.E. (2015). Present status of human papillomavirus vaccine development and implementation. Lancet Oncol..

[4] Singini M.G., Singh E., Bradshaw D., Chen W.C., Motlhale M., Kamiza A.B., de Villiers C.B., Muchengeti M., Mathew C.G., Newton R. (2022). Hpv types 16/18 l1 e6 and e7 proteins seropositivity and cervical cancer risk in hiv-positive and hiv-negative black south african women. Infect. Agents Cancer.

[5] Bruni L., Diaz M., Castellsague X., Ferrer E., Bosch F.X., de Sanjose S. (2010). Cervical human papillomavirus prevalence in 5 continents: Meta-analysis of 1 million women with normal cytological findings. J. Infect. Dis..

[6] Maitra A. (2019). Molecular envoys pave the way for pancreatic cancer to invade the liver. Nature.

[7] Massague J., Obenauf A.C. (2016). Metastatic colonization by circulating tumour cells. Nature.

[8] Hsiao Y.H., Lin C.W., Wang P.H., Hsin M.C., Yang S.F. (2019). The potential of chinese herbal medicines in the treatment of cervical cancer. Integr. Cancer Ther..

[9] Steeg P.S. (2006). Tumor metastasis: Mechanistic insights and clinical challenges. Nat. Med..

[10] Su S.C., Hsieh M.J., Yang W.E., Chung W.H., Reiter R.J., Yang S.F. (2017). Cancer metastasis: Mechanisms of inhibition by melatonin. J. Pineal Res..

[11] Su C.W., Lin C.W., Yang W.E., Yang S.F. (2019). Timp-3 as a therapeutic target for cancer. Ther. Adv. Med. Oncol..

[12] Marenholz I., Heizmann C.W., Fritz G. (2004). S100 proteins in mouse and man: From evolution to function and pathology (including an update of the nomenclature). Biochem. Biophys. Res. Commun..

[13] Ebralidze A., Tulchinsky E., Grigorian M., Afanasyeva A., Senin V., Revazova E., Lukanidin E. (1989). Isolation and characterization of a gene specifically expressed in different metastatic cells and whose deduced gene product has a high degree of homology to a ca2+-binding protein family. Genes Dev..

[14] Donato R., Cannon B.R., Sorci G., Riuzzi F., Hsu K., Weber D.J., Geczy C.L. (2013). Functions of s100 proteins. Curr. Mol. Med..

[15] Donato R. (2003). Intracellular and extracellular roles of s100 proteins. Microsc. Res. Tech..

[16] Boye K., Maelandsmo G.M. (2010). S100a4 and metastasis: A small actor playing many roles. Am. J. Pathol..

[17] Lee C.Y., Hsin M.C., Chen P.N., Lin C.W., Wang P.H., Yang S.F., Hsiao Y.H. (2022). Arctiin inhibits cervical cancer cell migration and invasion through suppression of s100a4 expression via pi3k/akt pathway. Pharmaceutics.

[18] Helfman D.M., Kim E.J., Lukanidin E., Grigorian M. (2005). The metastasis associated protein s100a4: Role in tumour progression and metastasis. Br. J. Cancer.

[19] Woo H.J., Kang H.K., Nguyen T.T., Kim G.E., Kim Y.M., Park J.S., Kim D., Cha J., Moon Y.H., Nam S.H. (2012). Synthesis and characterization of ampelopsin glucosides using dextransucrase from leuconostoc mesenteroides b-1299cb4: Glucosylation enhancing physicochemical properties. Enzym. Microb. Technol..

[20] Hou X.L., Tong Q., Wang W.Q., Shi C.Y., Xiong W., Chen J., Liu X., Fang J.G. (2015). Suppression of inflammatory responses by dihydromyricetin, a flavonoid from ampelopsis grossedentata, via inhibiting the activation of nf-kappab and mapk signaling pathways. J. Nat. Prod..

[21] Liao W., Ning Z., Ma L., Yin X., Wei Q., Yuan E., Yang J., Ren J. (2014). Recrystallization of dihydromyricetin from ampelopsis grossedentata and its anti-oxidant activity evaluation. Rejuvenation Res..

[22] Hou X., Tong Q., Wang W., Xiong W., Shi C., Fang J. (2015). Dihydromyricetin protects endothelial cells from hydrogen peroxide-induced oxidative stress damage by regulating mitochondrial pathways. Life Sci..

[23] Zhang Y., Ma J.N., Ma C.L., Qi Z., Ma C.M. (2015). Simultaneous quantification of ten constituents of xanthoceras sorbifolia bunge using uhplc-ms methods and evaluation of their radical scavenging, DNA scission protective, and alpha-glucosidase inhibitory activities. Chin. J. Nat. Med..

[24] Zhong Z.X., Qin J.P., Zhou G.F., Chen X.F. (2002). Experimental studies of hypoglycemic action on total flavone of ampelopsis grossedentata from guangxi. Zhongguo Zhong Yao Za Zhi.

[25] Xie J., Liu J., Chen T.M., Lan Q., Zhang Q.Y., Liu B., Dai D., Zhang W.D., Hu L.P., Zhu R.Z. (2015). Dihydromyricetin alleviates carbon tetrachloride-induced acute liver injury via jnk-dependent mechanism in mice. World J. Gastroenterol..

[26] Chen S., Zhao X., Wan J., Ran L., Qin Y., Wang X., Gao Y., Shu F., Zhang Y., Liu P. (2015). Dihydromyricetin improves glucose and lipid metabolism and exerts anti-inflammatory effects in nonalcoholic fatty liver disease: A randomized controlled trial. Pharm. Res..

[27] Zhang Q., Liu J., Liu B., Xia J., Chen N., Chen X., Cao Y., Zhang C., Lu C., Li M. (2014). Dihydromyricetin promotes hepatocellular carcinoma regression via a p53 activation-dependent mechanism. Sci. Rep..

[28] Chou C.H., Lu K.H., Yang J.S., Hsieh Y.H., Lin C.W., Yang S.F. (2021). Dihydromyricetin suppresses cell metastasis in human osteosarcoma through sp-1- and nf-κb-modulated urokinase plasminogen activator inhibition. Phytomedicine Int. J. Phytother. Phytopharm..

[29] Huang C.C., Su C.W., Wang P.H., Lu Y.T., Ho Y.T., Yang S.F., Hsin C.H., Lin C.W. (2022). Dihydromyricetin inhibits cancer cell migration and matrix metalloproteinases-2 expression in human nasopharyngeal carcinoma through extracellular signal-regulated kinase signaling pathway. Environ. Toxicol..

[30] Wang K., Yang S.F., Hsieh Y.H., Chang Y.Y., Yu N.Y., Lin H.W., Lin H.Y. (2018). Effects of dihydromyricetin on arpe-19 cell migration through regulating matrix metalloproteinase-2 expression. Environ. Toxicol..

[31] Zhang Q.Y., Li R., Zeng G.F., Liu B., Liu J., Shu Y., Liu Z.K., Qiu Z.D., Wang D.J., Miao H.L. (2014). Dihydromyricetin inhibits migration and invasion of hepatoma cells through regulation of mmp-9 expression. World J. Gastroenterol..

[32] Dahlmann M., Kobelt D., Walther W., Mudduluru G., Stein U. (2016). S100a4 in cancer metastasis: Wnt signaling-driven interventions for metastasis restriction. Cancers.

[33] Fei F., Qu J., Zhang M., Li Y., Zhang S. (2017). S100a4 in cancer progression and metastasis: A systematic review. Oncotarget.

[34] Kim B., Jung S., Kim H., Kwon J.O., Song M.K., Kim M.K., Kim H.J., Kim H.H. (2021). The role of s100a4 for bone metastasis in prostate cancer cells. BMC Cancer.

[35] Siegel R.L., Miller K.D., Jemal A. (2019). Cancer statistics, 2019. CA Cancer J. Clin..

[36] Zhou S., Peng F. (2020). Patterns of metastases in cervical cancer: A population-based study. Int. J. Clin. Exp. Pathol..

[37] Ullah A., Ullah N., Nawaz T., Aziz T. (2022). Molecular mechanisms of sanguinarine in cancer prevention and treatment. Anti-Cancer Agents Med. Chem..

[38] Xu Y., Wang S., Chan H.F., Lu H., Lin Z., He C., Chen M. (2017). Dihydromyricetin induces apoptosis and reverses drug resistance in ovarian cancer cells by p53-mediated downregulation of survivin. Sci. Rep..

[39] Fan T.F., Wu T.F., Bu L.L., Ma S.R., Li Y.C., Mao L., Sun Z.J., Zhang W.F. (2016). Dihydromyricetin promotes autophagy and apoptosis through ros-stat3 signaling in head and neck squamous cell carcinoma. Oncotarget.

[40] Chow K.H., Park H.J., George J., Yamamoto K., Gallup A.D., Graber J.H., Chen Y., Jiang W., Steindler D.A., Neilson E.G. (2017). S100a4 is a biomarker and regulator of glioma stem cells that is critical for mesenchymal transition in glioblastoma. Cancer Res..

[41] Ambartsumian N., Klingelhofer J., Grigorian M., Christensen C., Kriajevska M., Tulchinsky E., Georgiev G., Berezin V., Bock E., Rygaard J. (2001). The metastasis-associated mts1(s100a4) protein could act as an angiogenic factor. Oncogene.

[42] Saleem M., Kweon M.H., Johnson J.J., Adhami V.M., Elcheva I., Khan N., Bin Hafeez B., Bhat K.M., Sarfaraz S., Reagan-Shaw S. (2006). S100a4 accelerates tumorigenesis and invasion of human prostate cancer through the transcriptional regulation of matrix metalloproteinase 9. Proc. Natl. Acad. Sci. USA.

[43] Krausova M., Korinek V. (2014). Wnt signaling in adult intestinal stem cells and cancer. Cell. Signal..

[44] Holland J.D., Klaus A., Garratt A.N., Birchmeier W. (2013). Wnt signaling in stem and cancer stem cells. Curr. Opin. Cell Biol..

[45] Ring A., Kim Y.M., Kahn M. (2014). Wnt/catenin signaling in adult stem cell physiology and disease. Stem Cell Rev. Rep..

[46] Sawa M., Masuda M., Yamada T. (2016). Targeting the wnt signaling pathway in colorectal cancer. Expert. Opin. Targets.

[47] Fodde R., Smits R., Clevers H. (2001). Apc, signal transduction and genetic instability in colorectal cancer. Nat. Rev. Cancer.

[48] Yamaguchi H., Hsu J.L., Hung M.C. (2012). Regulation of ubiquitination-mediated protein degradation by survival kinases in cancer. Front. Oncol..

[49] Hsin M.C., Hsieh Y.H., Hsiao Y.H., Chen P.N., Wang P.H., Yang S.F. (2021). Carbonic anhydrase ix promotes human cervical cancer cell motility by regulating pfkfb4 expression. Cancers.

[50] Hsin M.C., Hsieh Y.H., Wang P.H., Ko J.L., Hsin I.L., Yang S.F. (2017). Hispolon suppresses metastasis via autophagic degradation of cathepsin s in cervical cancer cells. Cell Death Dis..

[51] Lin F.Y., Hsieh Y.H., Yang S.F., Chen C.T., Tang C.H., Chou M.Y., Chuang Y.T., Lin C.W., Chen M.K. (2015). Resveratrol suppresses tpa-induced matrix metalloproteinase-9 expression through the inhibition of mapk pathways in oral cancer cells. J. Oral Pathol. Med Off. Publ. Int. Assoc. Oral Pathol. Am. Acad. Oral Pathol..

[52] Su S.C., Yeh C.M., Lin C.W., Hsieh Y.H., Chuang C.Y., Tang C.H., Lee Y.C., Yang S.F. (2021). A novel melatonin-regulated lncrna suppresses tpa-induced oral cancer cell motility through replenishing prune2 expression. J. Pineal Res..

[53] Chung H.H., Hsieh M.J., Hsieh Y.S., Chen P.N., Ko C.P., Yu N.Y., Lin C.W., Yang S.F. (2021). The inhibitory effects of terminalia catappa l. Extract on the migration and invasion of human glioblastoma multiforme cells. Pharmaceuticals.

[54] Lu P.W., Chou C.H., Yang J.S., Hsieh Y.H., Tsai M.Y., Lu K.H., Yang S.F. (2022). Ho-3867 induces apoptosis via the jnk signaling pathway in human osteosarcoma cells. Pharmaceutics.

